# Assessing medication adherence in adults with asthma and its effect on rescue therapy for exacerbations

**DOI:** 10.3389/fphar.2025.1516062

**Published:** 2025-03-04

**Authors:** Yi Ling Eileen Koh, Kian Yong Kenny Chua, Ding Xuan Ng, Wai Keong Aau, Ngiap Chuan Tan

**Affiliations:** ^1^ SingHealth Polyclinics, Research, Singapore, Singapore; ^2^ National University of Singapore, Institute of System Science, Singapore, Singapore; ^3^ Defence Science and Technology Agency, Building and Infrastructure, Singapore, Singapore; ^4^ SingHealth-Duke NUS, Family Medicine Academic Clinical Program, Singapore, Singapore

**Keywords:** rescue therapy, Asthma Control test, proportion of days covered, inhalers, primary care

## Abstract

**Introduction:**

Adherence to prescribed inhaled controller medication is a determinant of asthma health outcomes. Traditional methods for assessing medication adherence (MA) can be challenging in real-world clinical settings. A new behavioral science approach presents opportunities to develop a novel MA assessment tool that also allows the prediction of acute asthma exacerbations. The current study aims to evaluate MA among adults with asthma based on their prescription collection behavior and its relationship with subsequent exacerbations.

**Method:**

This retrospective study was conducted on Asian adults with clinically diagnosed asthma and managed in public primary care clinics in Singapore from 2016 to 2023. Clinical data of patients, including socio-demographical, clinical (including Asthma Control Test scores), and prescription records were thoroughly examined to determine MA. The participants were stratified into the Full Collection (FC) group for those collections of prescribed asthma medication within a week; Partial Collection (PC) group for partial medication collection; No Collection (NC) group for no dispensation record within 1 year of the prescription date. The Proportion of Days Covered (PDC), defined as the proportion of days in which a patient gets access to the medication was determined to correlate with the prescription collection method. Multiple stepwise logistic regression was used to assess MA with rescue therapy (RT) occurrence as indicators of acute asthma exacerbations.

**Results:**

In this study, complete records of 13,482 patients were analyzed. The patients were categorized into three groups: FC (23.2%), PC (72.9%), and NC (3.9%) groups. Those who had PC or NC were more likely to have RT in the following year (19.5% and 9.4%, respectively), compared to FC (5.2%) group. Patients with RT demonstrated higher oral steroid dispensed compared to those without RT (mean (SD) of 319.7 (273.7) vs. 143.6 (175.8) compared to patients without RT (0.78 [0.26] vs. 0.81 [0.29]). Logistic regression analysis revealed that both patients from PC and NC groups had a greater probability of experiencing RT in the following year (partial: 2.364 (1.964–2.847), p < 0.001); no collection: 2.030 (1.318–3.127), p = 0.001). Lower minimum ACT scores (0.317 vs. 1.0) were noted for the PC group and an increase in minimal ACT score of 0.167 for every unit increase in PDC was also observed.

**Conclusion:**

Patients in the FC group exhibited higher MA and were less likely to receive RT due to their asthma exacerbations in subsequent years.

## 1 Introduction

Asthma, an inflammatory airway disease, can be life-threatening due to bronchoconstriction during an acute exacerbation. Inhaled corticosteroids (ICS) with or without Long-acting beta-agonists (LABA) are some of the evidence-based therapeutics to mitigate airway inflammation and mitigate asthma exacerbation risks (Cardet et al., 2023). ICS and LABA medications are prescribed by clinicians worldwide in a majority of healthcare settings. However, adherence to these inhaled medications might cause suboptimal outcomes globally (Gamble et al., 2009).

Assessment of medication adherence (MA) is the first pivotal step before considering appropriate and individualized solutions to address the underlying causes. Conventionally, clinicians prescribe different medications which the patients collect from the pharmacy after the consultation. Nonetheless, patients might opt to collect medications in totality, in installments, or not at all. Collection behavior for prescription is a surrogate marker of MA because a patient who collects all the medication presumably takes them regularly based on the prescribed doses.

The methodology of MA assessment of a patient is diverse, ranging from targeted history taking during a clinician-patient consultation, questionnaire-based inquiry, and prescription record review to the use of medical technology such as smart sensors embedded in the inhalers (Amber et al., 2023; Babel et al., 2021). Each method has its own strengths and limitations. Omission in the clinical approach occurs frequently due to limited consultation time, heavy patient load, oversight, and fatigue of clinicians. Incorporating routine use of questionnaires or reviews of manual or electronic prescription records necessitates trained healthcare workers, adequate logistic resources for decision support, clinical information, and redesign of care delivery system, which pose a multitude of challenges.

Prescription collection behavior is a useful tool to assess MA. Patients can choose to collect the medications in totality, in part, or not at all. A patient who collects all the medication is presumably taking them regularly based on the prescribed doses. Patients who filled their prescriptions partially over a stipulated period would suggest that they were not taking their medications regularly, resulting in a balance of medication in which a partially filled prescription was deemed adequate. Such an approach is strengthened by determining the Proportion of Days Covered (PDC) by the medication prescribed. PDC refers to the proportion of days in which a patient has access to the medication (Pharmacy Times, 2015; Shah et al., 2023).

The objective of the current research was to determine the medication adherence of adults with asthma based on their prescription collection behavior, and the proportion of days covered by their prescribed medication. It also aimed to evaluate the association between prescription collection behavior and the occurrence of rescue therapy (RT) as an indicator for acute asthma exacerbations in the subsequent year.

## 2 Methods

The study is based on retrospective electronic medical record (EMR) data from SingHealth Polyclinics, a public healthcare institution comprising a network of public primary care clinics in eastern Singapore.

### 2.1 Study sites and population

The study population comprises adults aged 21 years and above, who were diagnosed with asthma, and who were managed in eight public primary care clinics (polyclinics). These polyclinics managed more than two million patient visits in 2023. The institution's EMR system captures their demographic, clinical, treatment, prescription, and rescue therapy for acute asthma exacerbations at these polyclinics. The study population included individuals on inhaled controller medications with at least two asthma-related visits (medical consultations) recorded in the EMR within an eight-year period from 1 January 2016 to 31 December 2023. Ethics approval was obtained before the commencement of the study (SingHealth Centralized Institutional Review Board Ref No. 2023/2362).

### 2.2 Asthma medications

Medications listed in the institution formulary are dispensed from an in-house pharmacy in each polyclinic after prescription by a clinician (primary care physician or advanced practice nurse). The visit in which the consultation occurred, and the subsequent prescribed and dispensed medication are indexed with specific reference numbers in the EMR. The asthma medications in the formulary include inhaled corticosteroids (ICS) (Beclomethasone, Budesonide, and Fluticasone), inhaled combination medications (Fluticasone/Salmeterol and Budesonide/Formoterol), inhaled beta-agonist (Salbutamol) and oral prednisolone. The inhaled medications are available in different strengths and genres, such as meter-dosed inhalers, turbuhalers, and accuhalers (discus inhalers).

Patients pay for each medication according to the quantity dispensed from the pharmacy. Patients can opt to fill their prescriptions partially and collect the remaining medications later on. They can purchase the medication from private pharmacies in the community but a majority of the patients would obtain their subsidized medications at a lower cost at the polyclinic pharmacy.

### 2.3 Data management

Various categories of data including clinical notes, laboratory tests, prescribed and dispensed medications, and sociodemographic and financial status were extracted from their respective subject data warehouses, and transformed into actionable format via the ETL (Extract, Transform, Load) database function. Thereafter, the data were loaded into a single enterprise data repository known as Electronic Health Intelligence System (eHINTS) and subsequently retrieved by designated study team members.

The data included diagnosis codes of asthma and other comorbidities based on International Classification of Diseases version 10 (ICD-10); demographic variables such as age, gender, ethnicity, nationality, smoking status, and socioeconomic status; routine assessment of asthma status using Asthma Control test scores; clinical parameters such as height, weight; prescribed and dispensed inhaled and oral asthma medications, including daily doses and frequencies of such medications.

### 2.4 Definition of medication adherence

#### 2.4.1 By prescription collection behavior

Based on the prescription collection behavior, the patients were categorized into three groups: Full Collection (FC), Partial Collection (PC), and No Collection (NC).

a. FC: Patients collected the prescribed dose of medication within 1 week of the prescription date. Patients in this group reflect a high level of adherence to their prescribed treatment plan.
b. PC: Patients collected their medication more than 1 week after the prescription date but within 1 year, and included those who collected less than the prescribed quantity (with the balance of uncollected medications). The partial collection suggests suboptimal medication adherence by the patients in this group.
c. NC: Patients are classified in this category if no medication is dispensed within 1 year of the prescription date or within the period in between medical consultation, whichever is earlier. Patients in this group indicate poor adherence to the prescribed medication regimen.

Furthermore, the patients were grouped as FC or NC if they had records of only full collection or no collection throughout the 8-year period. However, those who had mixed permutation of full, partial, or no collection during this observation period, were classified under the PC group.

#### 2.4.2 Proportion of days covered

PDC is used as a surrogate marker for assessing MA. PDC is defined as the proportion of days in which a patient has access to the medication, considering early refills and shifting forward overlapping of medication supply; PDC ranges from 0 to 1, with 0 being poor adherence and 1 being good adherence (Pharmacy Times, 2015; Shah et al., 2023).

PDC is computed based on a calendar year timeframe. Therefore, medications with prescriptions that cross over into the next calendar year are split into two separate datasets, each assigned to the appropriate calendar year along with the corresponding medication supply information. Dosage for prescriptions and dispensations are merged by summing up the respective medications within the year to compute PDC.

To account for potential leftover medications from previous prescriptions, any remaining medication doses are carried over and added to the supply for the next visit of the patient. This ensures accurate PDC computation by considering the continuity of the medication in between visits which may straddle across a calendar year.

### 2.5 Outcome measures

The primary endpoint for the prediction model was defined as rescue therapy or RT (binary) in the treatment of acute asthma exacerbation and the frequency of RT in the polyclinic in the following calendar year. Rescue therapy refers to the inhalation of stipulated doses of reliever medication (salbutamol) based on the Institution’s approved care protocol. The protocol is implemented in each polyclinic to treat patients with acute asthma exacerbations. Another secondary outcome also includes the minimum ACT score.

### 2.6 Predictors of outcomes

The outcome predictors were selected based on theory, logic, and prior evidence, which are postulated to have a direct effect or mediate the outcomes in this study population:

1. Demographics and socioeconomic factors: age, gender, race, nationality, BMI, and smoking status, healthcare subsidies as surrogate indicators of their socioeconomic status.
2. Asthma control status based on self-reported outcomes using ACT scores, including its minimum and maximum scores, and the corresponding score for each of the five ACT questions.
3. Medications prescribed and dispensed during the observed period; the associated PDC, prescription collection groupings, and the number of polyclinic visits in each calendar year across the observation period.

### 2.7 Data preprocessing

**FIGURE 1:**
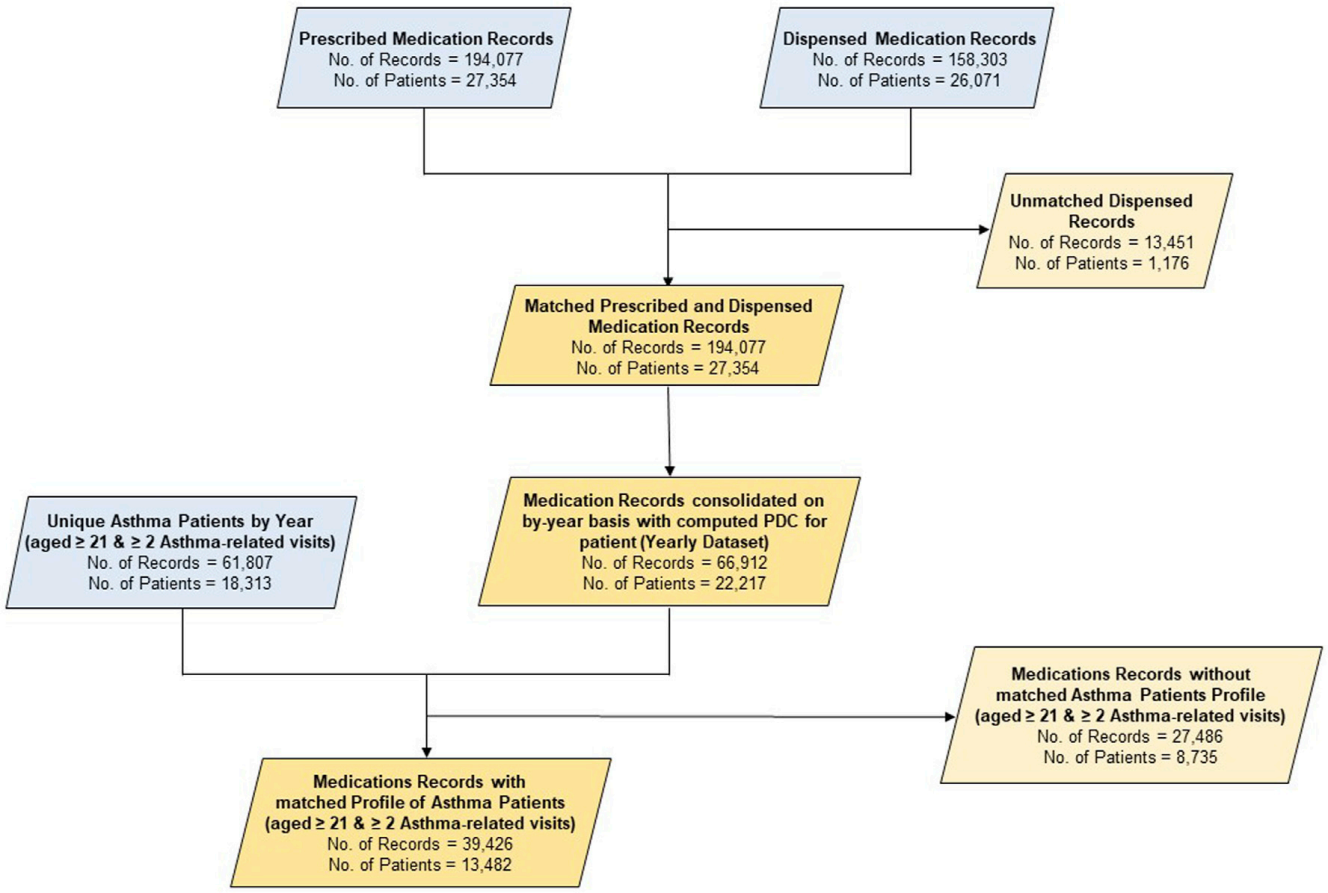
Data flow diagram. *#Note: Only inhalers (beclomethasone, budesonide and fluticasone, fluticasone/salmeterol and budesonide/formoterol, and salbutamol) and prednisolone tablets were included as medication for the study*.

An overview of the data preprocessing workflow is provided in Figure 1.

### 2.8 Linking prescribing and dispensing records

As the medication prescriptions can be actuated by the patient within a 1-year period after their visit, the corresponding dispensing record for that specific prescription may not be readily linked to the consultation visit reference number. For ease of computation, the dispensed record of the medication was linked to the most recent consultation visit in which the medication was prescribed.

### 2.9 Managing patients with incomplete data

Patients with missing EMR data on their smoking status, BMI, subsidies, comorbidities, RT, and ACT scores were considered to be void of any of these attributes or conditions.

### 2.10 Statistical analysis

Baseline characteristics such as age, gender, race, nationality, BMI, and smoking status were taken at index visits in the study period, while data on medication, ACT scores, and comorbidities were recorded at any point during the study period. Continuous variables were expressed as mean ± SD and categorical variables were expressed in terms of frequency and percentages.

Chi-square tests were performed to examine the association between the prescription collection behavior and the profile of the patients and to assess the prescription collection behavior and the categorical demographics variables like gender, race, nationality, smoking status, subsidies, medications, and comorbidities. Shapiro–Wilk test was used to evaluate the normality of the continuous data such as age, BMI, PDC, ACT scores, and number of RT visits. The association of normally distributed continuous variables with prescription collection behavior was determined using ANOVA tests while Kruskal-Wallis tests were used for the non-parametric continuous variables.

To assess multicollinearity between variables, the Variance Inflation Factor (VIF) was calculated for each predictor variable. Variables with a VIF of more than 10 were omitted due to the potential multicollinearity.

For the outcome on binary RT, forward stepwise logistic regression was performed to obtain the adjusted odds ratios and 95% confidence intervals. Contrastively, forward stepwise linear regression was employed to evaluate the association between MA and minimum ACT score.

Scatterplots were also plotted to examine the association between prednisolone dosage and salbutamol dosage with the number of RT, and Spearman’s correlation was reported.

A p-value of less than 0.05 is considered statistically significant. All statistical analyses were generated using software Python version 3.11.5 and IBM SPSS version 29.0.

## 3 Results

### 3.1 Baseline characteristics of the study population

A total of 13,482 patients with asthma were included in the analysis (Table 1); of them, 2132 (15.8%) had undergone at least 1 RT in the following year. The study population had a mean age of 54.4. Furthermore, 57.4% of them were female individuals, half of them were Chinese, and a quarter of them were Malay. Indians and other nationality patients contributed to the remaining 25% of the patients' population. Three groups of patients were formed: 23.2% had FC, 72.9% had PC, and 3.9% had NC in the entire study period. Patients from the PC or NC groups were more likely to have RT in the following year (19.5% and 9.4%), compared to FC (5.2%).

**TABLE 1:** Baseline characteristics of the study population and their association of medication adherence status and RT in the following year.

	Total	Occurrence of RT in the following year		
		No	Yes	p-value
Total	13,482 (100)	11,350 (84.2)	2,132 (15.8)	
Prescription collection behavior				<0.001
Full collection	3,127 (23.2)	2,965 (94.8)	162 (5.2)	
Partial collection	9,831 (72.9)	7,910 (80.5)	1921 (19.5)	
No collection	524 (3.9)	475 (90.6)	49 (9.4)	
PDC, mean(SD)	0.8 (0.3)	0.81 (0.29)	0.78 (0.26)	<0.001
Age, year, mean(SD)	54.4 (17.4)	53.9 (17.8)	56.9 (15.4)	<0.001
BMI, kg/m^2^, mean(SD)	27.2 (6.1)	27 (6.1)	27.8 (6.3)	<0.001
Gender				0.470
Female	7,741 (57.4)	6,532 (84.4)	1,209 (15.6)	
Male	5,741 (42.6)	4,818 (83.9)	923 (16.1)	
Race				<0.001
Chinese	6,888 (51.1)	5,871 (85.2)	1,017 (14.8)	
Malay	3,549 (26.3)	2,967 (83.6)	582 (16.4)	
Indian	1824 (13.5)	1,486 (81.5)	338 (18.5)	
Others	1,221 (9.1)	1,026 (84)	195 (16)	
Nationality				0.999
Singaporean	13,071 (97)	11,004 (84.2)	2067 (15.8)	
Non-Singaporean	411 (3)	346 (84.2)	65 (15.8)	
Smoking				0.018
Smoker	115 (0.9)	106 (92.2)	9 (7.8)	
Non-smoker	13,367 (99.1)	11,244 (84.1)	2,123 (15.9)	
Subsidy status				0.320
Subsidized	8,745 (64.9)	7,342 (84)	1,403 (16)	
Non-subsidized	4,737 (35.1)	4,008 (84.6)	729 (15.4)	
On financial assistance				<0.001
Yes	769 (5.7)	614 (79.8)	155 (20.2)	
No	12,713 (94.3)	10,736 (84.4)	1977 (15.6)	
Total visits in the year, mean(SD)	3.3 (1.6)	3.2 (1.5)	4.3 (2)	<0.001
ICS brand change	75 (0.6)	59 (78.7)	16 (21.3)	0.189
Combination med brand change	356 (2.6)	222 (62.4)	134 (37.6)	<0.001
Switching brand between ICS and combination med	1,234 (9.2)	867 (70.3)	367 (29.7)	<0.001
Prescribed ICS medication	6,925 (51.4)	5,896 (85.1)	1,029 (14.9)	0.002
Prescribed combined controller medication	8,488 (63)	6,818 (80.3)	1,670 (19.7)	<0.001
Prednisolone dosage, mg, mean (SD)	171.4 (204.9)	143.6 (175.8)	319.7 (273.7)	<0.001
Salbutamol inhalers dispensed, mean (SD)	0.7 (1.5)	0.6 (1.4)	1.3 (1.8)	<0.001
Min. ACT total score, mean (SD)	17.7 (4.4)	18.3 (4.2)	14.9 (4.3)	<0.001
Min. ACT Q1 (impact on daily activities) score, mean (SD)	3.9 (1)	4 (1)	3.3 (1.1)	<0.001
Min. ACT Q2 (frequency of shortness of breath) score, mean (SD)	3.6 (1.2)	3.8 (1.1)	3 (1.2)	<0.001
Min. ACT Q3 (nighttime symptoms) score, mean(SD)	3.5 (1.4)	3.6 (1.3)	2.7 (1.3)	<0.001
Min. ACT Q4 (use of rescue inhaler) score, mean (SD)	3 (1.3)	3.1 (1.3)	2.3 (1.2)	<0.001

### 3.2 Association between medication and rescue therapy

Patients with RT in the following year showed lower PDC compared to those without RT [0.78 (0.26) vs. 0.81 (0.29)] (Table 1). The people who had RT in the following year were also more likely to visit the polyclinic as compared to those who did not present RT (mean of 4.3 vs. 3.2).

In terms of oral steroid dispensed, the people who had RT in the following year had a mean of 319.7 (273.7) prednisolone dosage dispensed, compared to a mean of 143.6 (175.8) in the non-RT group. Patients with Salbutamol dispensed were also higher in number in the RT group compared to the non-RT group (1.3 vs. 0.6).

### 3.3 Association between medication collection status and rescue therapy

Table 2 Large number of patients in the FC group had the lowest mean age (46.2 years), thereby indicating a younger population, compared to over 50 years in the PC and NC groups. They also revealed the best medication adherence, with a PDC score of 1.00. However, the patients in the PC group had a mean PDC of 0.80, and the NC group had a notably lower mean PDC of 0.

**TABLE 2:** Profile of patients by their prescription collection status.

Characteristic	All patients	Full collection	Partial collection	No collection	P value
Total	13,482 (100.0)	3,127 (23.2)	9,831 (72.9)	524 (3.9)	
Age, year mean (SD)	54.4 (17.4)	46.2 (17.5)	57.1 (16.5)	52.6 (18.8)	<0.001
BMI, kg/m^2^ mean (SD)	27.2 (6.1)	27.2 (6.5)	27.1 (6.0)	27.3 (6.5)	<0.001
PDC mean (SD)	0.8 (0.3)	1.0 (0.0)	0.8 (0.3)	0.0 (0.2)	<0.001
Total visits in year mean (SD)	3.3 (1.6)	2.3 (1.3)	3.7 (1.6)	2.8 (1.6)	<0.001
Gender					<0.001
Female	7,741 (57.4)	1718 (22.2)	5,736 (74.1)	287 (3.7)	
Male	5,741 (42.6)	1,409 (24.5)	4,095 (71.3)	237 (4.1)	
Race					<0.001
Chinese	6,888 (51.1)	1,331 (19.3)	5,313 (77.1)	244 (3.5)	
Malay	3,549 (26.3)	1,045 (29.4)	2,366 (66.7)	138 (3.9)	
Indian	1824 (13.5)	434 (23.8)	1,305 (71.5)	85 (4.7)	
Others	1,221 (9.1)	317 (26.0)	847 (69.4)	57 (4.7)	
Smoking					0.003
Smoker	115 (0.9)	38 (33.0)	75 (65.2)	2 (1.7)	
Non-smoker	13,367 (99.1)	3,089 (23.1)	9,756 (73.0)	522 (3.9)	
Cost of medication					<0.001
Subsidized	8,745 (64.9)	1874 (21.4)	6,536 (74.7)	335 (3.8)	
Non-subsidized	4,737 (35.1)	1,253 (26.5)	3,295 (69.6)	189 (4.0)	
On financial assistance					<0.001
Yes	769 (5.7)	206 (26.8)	529 (68.8)	34 (4.4)	
No	12,713 (94.3)	2,921 (23.0)	9,302 (73.2)	490 (3.9)	

### 3.4 Multiple regression analysis for rescue therapy and ACT with medication adherence

Model 1 given in Table 3 shows that partial and no prescription collection are more likely to have RT in the following year (partial: 2.364 (1.964–2.847), p < 0.001, no collection: 2.030 (1.318–3.127), p = 0.001). Model 2 shows an increase of 0.307 in ACT score when there is partial collection, and a 0.167 increase in ACT score when PDC increases by 1 unit.

**TABLE 3:** Association of medication adherence with rescue therapy occurrence and minimum total ACT score.

	Model 1^a^		Model 2^b^	
	Adjusted OR (95% CI)	p-value	Beta coefficient (95% CI)	p-value
Prescription collection				
Full collection	1	—	Reference	—
Partial collection	2.364 (1.964–2.847)	<0.001	0.317 (0.274–0.36)	<0.001
No collection	2.03 (1.318–3.127)	0.001	0.106 (−0.004–0.215)	0.058
PDC	1.086 (0.859–1.372)	0.490	0.167 (0.095–0.239)	<0.001

^a^ Model 1: Rescue therapy (yes/no) in the following year as outcome, adjusted for age, BMI, gender, smoking status, prescription collection status, PDC, combined controller prescribed, prednisolone prescribed, salbutamol prescribed, minimum ACT (Q1-Q4), dispensed prednisolone dosage, and dispensed salbutamol using stepwise multiple logistic regression.

^b^ Model 2: Minimum total ACT, score as outcome, adjusted for age, race, on financial assistance, prescription collection status, PDC, combined controller prescribed, prednisolone prescribed, salbutamol prescribed, minimum ACT (Q1-Q5), dispensed prednisolone dosage, dispensed salbutamol, total polyclinic visits in the year, ICS prescribed, and switched ICS, in the year using stepwise multiple linear regression.

### 3.5 Correlation between the frequency of rescue therapy and oral steroids

**FIGURE 2:**
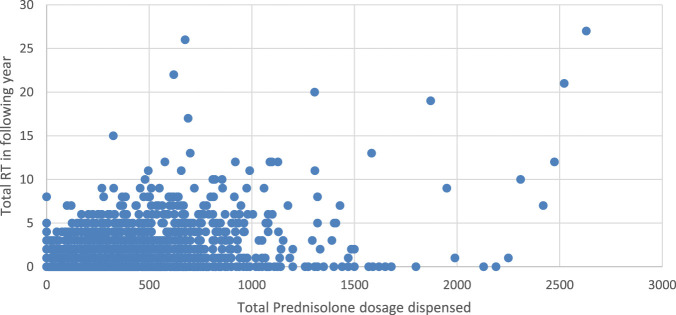
Scatterplot showing an association between RT and prednisolone dosage dispensed.

**FIGURE 3:**
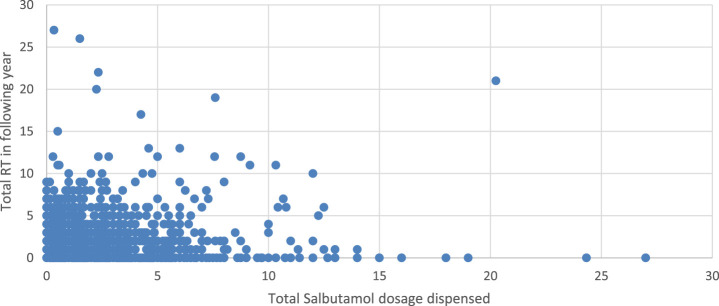
Scatterplot showing an association between RT and salbutamol dosage dispensed.

Figure 2 Higher doses of Prednisolone were generally associated with an increased number of RT, Spearman’s correlation is 0.315, p < 0.001 between RT and dispensed prednisolone. Figure 3 depicts an increased number of RT with higher dosages of salbutamol dispensed.

## 4 Discussion

Asthma exacerbations are defined as a worsening of asthma symptoms that require medical intervention. They constitute a major cause of morbidity and healthcare utilization, thus highlighting the need for effective management strategies (Global Initiative for Asthma GINA, 2024). Inhaled medications, including ICS and bronchodilators, are fundamental in managing asthma symptoms and improving respiratory function. Long-acting beta-agonists (LABAs), when used in combination with ICS, provide bronchodilation and help improve airflow by relaxing the smooth muscles surrounding the airways (Papi et al., 2020). Inhaled medications offer rapid relief of bronchoconstriction and long-term control when used consistently (Global Initiative for Asthma GINA, 2024). Hence they are more efficient in managing asthma symptoms.

Despite the clear benefits of inhaled medications, adherence remains a significant challenge in asthma management. The effectiveness of different categories of inhaled medications for asthma pharmacotherapy varies but failure to use them regularly will significantly raise the risk of future asthma exacerbations (Yamasaki et al., 2024). To our knowledge, leveraging prescription collection behavior to assess MA is novel in this study, with no precedent to benchmark from published literature. The results show that more than 7 out of 10 patients who collected their asthma medications partially, were likely to reflect their poor medication adherence. It is further substantiated by the computation of PDC, with a score of 0.8, reflecting that patients may not use their medications up to 20% of the time. More rigorous endeavors are needed by healthcare professionals to review the treatment adherence or even re-examine the diagnosis when red flags are raised when their patients choose to collect their medication partially or completely default their prescription.

Adults who collected the medications partially tended to be significantly older, female individuals, and Chinese, receiving subsidized medication and conversely, fewer received financial assistance from the official agency (Table 2). It seems that the cost of the medication is less likely to be a barrier to prescription collection as the inhalers are largely dispensed at subsidized prices to local citizens or permanent residents and financial assistance is readily available to those who could not afford the medications. Older adults may have more co-morbidities and are often treated with other medications aside from inhalers. The resultant polypharmacy is postulated to jeopardize MA. It is suggested that more qualitative research be performed in the future to dive deep into the impact of the socio-demographic factors of these adults on their prescription collection behavior.

Significant association exists between the prescription collection behavior of these adults and their asthma outcome and morbidity (Table 3). Regardless of data shown in Model 1 or 2, those with PC or NC were at higher risks of RT in the following year. Most current models leveraged on data relating to emergency department (ED) visits or hospitalization as the definition for asthma exacerbation, but few have looked at RT as the outcome and in the context of primary care-based asthma management. A systematic review pooled the performance of asthma exacerbation prediction models; of them, 73% were based on hospitalization and ED visits (Xiong et al., 2023). However, asthma is largely managed in primary care. It allows opportunities for pharmacists and clinicians to explore and address their MA as their prescription collection behavior can be readily identified in primary care or community pharmacies.

Prescribing controller medication such as ICS or combination medication (ICS + Long-Acting Beta Agonist or LABA) or switching ICS brand or between ICS and combination medication) had a significant effect on their asthma control status based on the total or individual item score of the Asthma Control test, and the risk of RT in the following year (Table 1). More relievers such as Salbutamol inhalers were inevitably dispensed for those with RT, which is expected based on clinical practice guidelines in treating acute asthma. It is vital to counsel patients on adherence to ICS or combination medication after they are identified with partial prescription collection.

The NC group is far smaller compared with those in the FC and PC groups. It is a subset of the population who are often overlooked unless deliberate efforts are devoted to comparing prescribed and dispensed medications. The current electronic prescription system has yet to automate the computation of such differences. However, their asthma health status was not drastically compromised with low RT risk despite the absence of medication. This group may compromise those with a provisional diagnosis of asthma pending their scheduled spirometry or be collecting their medication from other healthcare providers, including those who were managed by pulmonologists from hospitals.

A paradigm shift in asthma management can emerge in which asthma prescription collection behavior by adults with asthma at the primary care or community pharmacy triggers alerts to pharmacists and clinicians to investigate their MA. Healthcare professionals can explore the reasons for the partial collection of asthma exacerbation occurrences and frequencies during wide-ranging consultations to review their asthma pharmacotherapy and MA. Improving adherence through multidisciplinary primary healthcare professionals, such as pharmacist-patient consultations is another strategy to optimize asthma care (Makhinova et al., 2022). Motivation for self-management, seeking asthma knowledge, improving attitudes, and having a positive trusting relationship with the healthcare provider have been shown to be pivotal factors in enhancing MA (Blake, 2017). Delegating more frequent review of asthma adherence to pharmacists, or other allied health providers who could provide adherence counseling would be a practical alternative (Soones et al., 2017).

In recent years, smartphone-enabled sensors, electronic trackers or reminders, and simplified regimens showed better adherence, reduced rescue inhaler use, and improved Asthma Control Scores (Foster et al., 2014; Normansell et al., 2017). Considering the results of this study, the institution has implemented an automated digital interface to rapidly identify patients who collect their medications entirely or partially when they present themselves at the polyclinic pharmacy. The next step is to design a clinical trial involving such a new care model, especially in a resource-poor community, to evaluate its effectiveness in rapidly identifying and targeting adult patients who collect their medications partially. This measure aims to assess the underlying reasons for the partial collection, including those relating to MA, and individualize the solution and action plan to address any specific deficiencies during the asthma care delivery in the polyclinics.

### 4.1 Limitations

The study has certain limitations such as the absence of detailed information about patients’ failure to collect their medications entirely, if at all. The results are also based on the likely incorrect assumption that those who collected their medications were using them according to their prescriptions and were proficient in using the various types of inhalers. Patients using the budesonide/formoterol intermittently in the Single Maintenance And Reliever Therapy (SMART) way could be classified in the PC group, but this is an evidence-based therapeutic approach in the clinical practice guidelines.

The RT was based solely on the EMR in the institution. Patients could seek treatment for their asthma exacerbation by other healthcare providers in the community and in the emergency units of hospitals, which were not included in this retrospective study. Exacerbations can be aborted by patients themselves as they were provided with an asthma action plan to initiate their rescue therapy on their own outside of healthcare facilities. The dose of dispensed prednisolone (Table 1) seems staggering even in the RT group because oral steroid is prescribed as a standby medication for self-management to mitigate asthma attacks.

## 5 Conclusion

Overall, it can be concluded that patients who collected full prescriptions of their asthma medications exhibited greater MA and had fewer RT for asthma exacerbations in the following year.

## Data Availability

The datasets presented in this article are not readily available because of privacy and confidentiality concerns but are available from the corresponding author on reasonable request. Requests to access the datasets should be directed to tan.ngiap.chuan@singhealth.com.sg.
